# A Social Virtual Reality-Based Application for the Physical and Cognitive Training of the Elderly at Home

**DOI:** 10.3390/s19020261

**Published:** 2019-01-10

**Authors:** Sara Arlati, Vera Colombo, Daniele Spoladore, Luca Greci, Elisa Pedroli, Silvia Serino, Pietro Cipresso, Karine Goulene, Marco Stramba-Badiale, Giuseppe Riva, Andrea Gaggioli, Giancarlo Ferrigno, Marco Sacco

**Affiliations:** 1 Istituto di Sistemi e Tecnologie Industriali Intelligenti per il Manifatturiero Avanzato, Consiglio Nazionale delle Ricerche, 20133 Milano, Italy; vera.colombo@stiima.cnr.it (V.C.); daniele.spoladore@stiima.cnr.it (D.S.); luca.greci@stiima.cnr.it (L.G.); marco.sacco@stiima.cnr.it (M.S.); 2 Dipartimento di Elettronica, Informazione e Bioingegneria, Politecnico di Milano, 20133 Milano, Italy; giancarlo.ferrigno@polimi.it; 3 Applied Technology for Neuro-Psychology Lab, I.R.C.C.S. Istituto Auxologico Italiano, 20149 Milano, Italy; e.pedroli@auxologico.it; 4 Department of Psychology, Università Cattolica del Sacro Cuore, 20123 Milano, Italy; silvia.serino@unicatt.it (S.S.); pietro.cipresso@unicatt.it (P.C.); giuseppe.riva@unicatt.it (G.R.); andrea.gaggioli@unicatt.it (A.G.); 5 Department of Geriatrics and Cardiovascular Medicine, I.R.C.C.S. Istituto Auxologico Italiano, 20149 Milano, Italy; goulene@auxologico.it (K.G.); stramba_badiale@auxologico.it (M.S.-B.)

**Keywords:** ageing, virtual reality, social media, collaboration, competition

## Abstract

Frailty is a clinical condition affecting the elderly population which results in an increased risk of falls. Previous studies demonstrated that falls prevention programs are effective, but they suffer from low adherence, especially when subjects have to train unsupervised in their homes. To try to improve treatment adherence, virtual reality and social media have been proposed as promising strategies for the increase of users’ motivation and thus their willingness to practice. In the context of smart homes, this work presents SocialBike, a virtual reality-based application aimed at improving the clinical outcomes of older frail adults in their houses. Indeed, SocialBike is integrated in the “house of the future” framework and proposes a Dual Task training program in which the users are required to cycle on a stationary bike while recognizing target animals or objects appearing along the way. It also implements the possibility of training with other users, thus reducing the risk of social isolation. Within SocialBike, users can choose the multiplayer mode they prefer (i.e., collaborative or competitive), and are allowed to train following their own attitude. SocialBike’s validation, refinement, and business model are currently under development, and are briefly discussed as future works.

## 1. Introduction

In recent years, there has been a notable growth of the ageing population. According to the World Health Organization, the proportion of people older than 65 years-old is increasing faster than that of all the other age groups, and is expected to triple to 1.5 billion by the mid-21st century [1,2].

Ageing is a process that leads to the structural and functional deterioration of many physiological systems, even in the absence of a specific pathology [3]. The age-related loss of functional properties yields a decreased adaptability to internal and external stress, thus leading to a condition of increased vulnerability to disease and mortality [4]. This situation causes an increasing economic and social burden for both countries and care-givers [5], and calls for the development of new technological solutions supporting ageing people and, in general, people with disabilities during their activities of daily living (ADLs) [6], especially in their own homes. 89% of older adults, in fact, prefer to stay in the comfort of their own homes, but the costs of nursing home care are often not bearable to ensure that they can safely age in place [7]. Ambient Assisted Living (AAL) is a research paradigm born to try answering to these needs. In detail, AAL exploits Ambient Intelligence (AmI, e.g., information technology solutions that are sensitive, adaptive, and responsive to human needs [8,9]) with the aim of preventing and managing diseases, and improving the wellness and health conditions of older adults. AAL includes tools such as medication and events reminders [10], monitoring systems (e.g., for fall detection [11]), assistive devices to help mobility and autonomy in ADLs [12,13], and technologies to promote a healthier lifestyle and socially-active ageing [14]. 

In the context of AAL, the Future Homes for Future Communities (FHfFC) project [15] aims at creating the “house of the future”, by integrating several technological solutions to promote social participation for house dwellers, enhance their safety, allow them to continue rehabilitative treatments at home after de-hospitalization, and support their ADLs through robotic assistive technologies. This smart home leverages on semantic web technologies to provide a coherent representation of several domains of knowledge (dwellers’ health statuses, devices deployed in the house, comfort metrics, etc.,) and can be controlled using the Home Interactive Controller (HIC) [16]. The HIC consists of an interface that allows the inhabitants to control several aspects of indoor comfort (e.g., temperature, humidity rate, illuminance, CO_2_ concentration) and assists them in the performance of activities of daily living, such as the preparation of a meal. The HIC can be used via a tablet, or on any flat surface by using an interactive finger-touch projector (e.g., Epson EB-695WI, implemented in the FHfFC living lab, Lecco, Italy). Finally, the HIC can be used to enable specific FHfFC smart home services, such as SocialBike. SocialBike—one of the outcomes of FHfFC project—is a social virtual reality-based application, developed on the basis of an already clinically tested system [17], for the implementation of a home-based Dual Task (DT) intervention to prevent falls in frail elderly. 

Frailty is a common pathological condition among the elderly. It affects 4–59% of people older than 65 years-old [18], and manifests with symptoms in gait, mobility, balance, and cognition [19]. 

Among frail patients, falls are one of the most critical issues, and are the major cause of injuries [20,21]. Episodes of falls are critical in the ageing population, since they often lead to loss of autonomy, adverse psycho-social problems [22], and increased risk of isolation and death [23]. 

Therefore, several geriatric interventions have been developed either to improve clinical outcomes for frail older adults or to try to prevent the onset of frailty [24,25]. Among these, Dual Task (DT) training has recently been proposed as a potentially effective strategy for reducing the risk of falling in the elderly population [26]. DT consists of the concurrent execution of a cognitive and a motor task, which has been demonstrated to lead frail elder individuals to a reduced performance in either the motor or cognitive executions, or even in both [27,28,29]. Since many ADLs require the ability to maintain balance while performing a concurrent motor or cognitive task, and falls occur more frequently when the subject is performing two interfering activities simultaneously, training using DT appears to be a helpful solution for the prevention of falling episodes. 

Fall prevention programs have also been recently implemented in home-based settings [30,31]. This approach has emerged as a potentially cost-effective means to improve the clinical outcomes of frail elderly, and thus reduce the burden of frailty for both the individuals and society [32]. On the other hand, undergoing home-based intervention may reduce the already-limited possibilities of social participation in the community that frail homebound elderlies usually have [33]. Therefore, it is crucial that interventions developed for the improvement of frailty take into account the social aspect, as it is a key element to promote wellbeing and to prevent the onset of social isolation, depression, and low quality of life [33,34]. 

For these reasons, the SocialBike application proposes a DT training, but also offers the end-users the possibility to exercise while talking via chat to other elderlies, so that their motivation and their social participation can be promoted too.

## 2. Related Work

Poor postural control and muscle weakness are among the major risk factors for falls. Ageing negatively influences the capabilities of maintaining a stable posture and recovering after a perturbation, because it leads to reduced visual, proprioceptive, and vestibular sensitivity. Different solutions have been implemented during the last years to reduce the risk of falling in elderly or frail populations. 

Home modifications, for instance, are often exploited to prevent falls in the elderly with a history of fallings [35]. These occupational interventions include hazard identification, structural indoor and outdoor changes, and provision of assistive technology and assistive devices [35]. Some of these modifications also include AmI technologies, though they are still not considered as standard practice and the attitudes of the elderly toward these technologies still have to be clarified [36]. 

Other solutions to prevent falls focus on physical training [37,38] or on a combination of home modifications and exercise programs. To try to counteract the age-related reduced perception, exercise-based interventions aim at targeting balance, muscle strength, flexibility, and endurance [39]. However, these programs often suffer from low therapy adherence, especially when the subject has already reported one or more falling episodes [38,40] or when he/she has to practice at home, not supervised by an expert therapist [41]. 

Since exercise therapy has been proven to be effective when the user sticks to the program, researchers are currently working to promote motivation to train among elderly people. In this context, “exergames” (exercise + game) emerged as a promising solution to stimulate adherence, because of their ludic component, and they can also enable the elderly to train at home. Exergames enabled by the use of virtual reality (VR) allow for the creation of ecological and controlled environments specifically designed to challenge the users and increase their motivation, thus leading to higher adherence to treatment [42]. In general, exergames require the user to perform stepping movements, weight-shifting, reaching movements, and/or aerobic exercises [42], and allow for the introduction of different cognitive tasks to be performed concurrently while doing the physical activities, thus easing the process of creating DT training programs. 

Another aspect that is emerging to stimulate user engagement and motivation is the possibility of training with or challenging another person. Multiplayer games have been proven to have positive effects on the task performance, as well as the willingness to play [43]. Therefore, designing rehabilitative applications to include this aspect may also act as an effective strategy to increase the adherence to the intervention, thus improving the long-term effectiveness of the program. 

Multiplayer games are characterized by the way in which the users play. In some scenarios, players compete against each other, whereas in other cases they play in teams, collaborating with other team members [44]. 

In general, it has been shown that performing physical activity in a competitive situation induces young subjects to reach higher levels of energy expenditure [43,45,46]. A study conducted using a hula-hoop exercise (with the Nintendo Wii Board) revealed that college students performed better when playing against virtual competitors, independent of their levels of competitiveness [47]. 

Although these behaviors may be different when comparing the attitudes of young and older populations, the same tendency was also found in a study enrolling elderlies: Competitive subjects were found to significantly increase their pedaling intensity when asked to compete against a virtual avatar [48]. On the other hand, other studies demonstrated that the performances of non-competitive elderlies were not influenced by the avatar presence. In two cases, the non-competitive elderlies had no improvements with respect to the solo performance [48,49]. In another case, competition was even demonstrated to decrease the motivation of players who were non-competitive, making them more likely to abandon the program [47]. Thus, in cases of non-competitive subjects, proposing collaborative scenarios may be the key for enhancing their motivation. Indeed, some studies showed that collaborative games have a positive influence on motivation and self-efficacy and, as a consequence, on the adherence to physical activity [43,50]. Another interesting result is that collaborative modes are more effective when all the players are human, rather than virtual, because feeling useful seems to be as important a motivator as getting help [51,52].

All these findings support the importance of tailoring the participation in the programs in competitive or collaborative scenarios, based on the subject’s personality and attitude. SocialBike has been developed taking this aspect into account: It provides the users with the possibility of choosing between a collaborative or competitive experience at the beginning of each session. 

## 3. A Social Application for Dual Task Training

In 2017, Positive Bike, a system employing a cycle ergometer to provide frail elderlies or frequent fallers with DT training, was developed as a result of a focus group involving both technical (engineers, IT scientists, and designers) and clinical experts (psychologists, physical therapists, and clinicians) [23]. The stationary bike, placed inside a Cave Automatic Virtual Environment (CAVE), allowed the users to train their cognitive abilities while cycling by recognizing target objects appearing along the route. 

This setup was the first one exploiting a cycle ergometer to provide the physical training during a DT task. There are several reasons underlying the use of a cycle ergometer instead of a treadmill (i.e., the most exploited equipment for the provision of DT training). First, cycling exercises have been proven to improve balance, center of pressure displacement control, and gait, thus resulting in a significant reduction of the fall risk [28,29]. The pattern of cycling, in fact, shares many characteristics with the gait pattern: Both are cyclical, and both foresee the alternative occurrence of flexion and extension movements in the involved joints (i.e., ankle, knee, and hip) [30,31,32]. Finally, with respect to the use of the treadmill, the employment of a stationary bike not only allows for controlling the workload, but also reduces the risk of injuries while training [33], thus providing a safer training environment, which is especially fundamental for home-based interventions dedicated to the elderly.

In 2018, SocialBike was designed by the same focus group. Though it was grounded on the same rehabilitative paradigms, this application was modified and improved for home-based exploitation. Indeed, SocialBike resembles the features of Positive Bike, and it implements some modifications to permit home-based use (e.g., the use of a normal screen, a less-expensive technology, instead of a CAVE) and to promote the social participation of the elderly (i.e., the multiplayer component).

The training takes place in a virtual park in which the player has to cycle. The scenario foresees the typical elements of a park environment (trees, grass, lakes, etc.) plus specific elements by the side of the road, which will be referenced as *target* objects, i.e., objects or animals that the subjects have to recognize as a part of the cognitive task.

The physical activity consists of pedaling for 20 min on a cycle ergometer while keeping the speed constant between 60 and 70 revolutions per minute (RPM). In order to maintain a constant level of effort for all users independently from their physical status, the workload of the cycle ergometer is adjusted according to the heart rate of the subject. In particular, a digital controller allows for the variation of the workload to maintain the heart rate at around 65% (HR_target_) of the maximum value (HR_max_), estimated according to Equation (1):

HR_target_ = 0.65 × HR_max_ = 0.65 × (208 − 0.7 × age) (1)

The choice of these parameters was made according to physicians’ knowledge, and was based on the results of previous studies performed on elderlies with minor cognitive impairments [53]. Sixty-five percent, in particular, was defined by the American College of Sports Medicine (ACSM) as corresponding to the minimum effort capable of eliciting an increase in the user’s cardiorespiratory fitness, i.e., the minimum effort that produces a training effect [54]. This value constitutes the default training intensity and each time a new user is created, 65% is automatically assigned. However, in the case of subjects that are either particularly fit or debilitated, the physician may choose to increase or decrease this value accordingly.

All the players in SocialBike are forced to train while keeping their heart rate constant. This allows for the exerted physical effort to be made comparable for everybody, thus avoiding more athletic players leaving the others behind and weaker subjects feeling frustrated by the others’ performances. Moreover, to avoid the arousal of anxiety and frustration, data regarding the physical exercise are kept “private” and are hidden to the other players.

**Figure 1:**
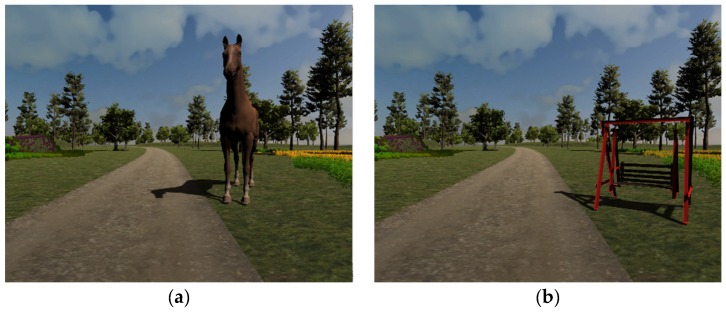
Examples of the two types of targets: A horse (**a**) and a colored swing (**b**).

The cognitive task consists of identifying a series of target objects appearing by the side of the pathway, according to two different criteria [17]. If players are exercising using animals, at the beginning of the exercise, each player is assigned a letter and he/she is asked to identify all the animals whose name starts with that letter (e.g., for the letter “d”, “dog” is a target, whereas “horse” is not). Instead, if the target objects are street furniture, each player is assigned a color, as shown in Figure 1.

The Dual Task training can be performed while either collaborating or competing. In the first case, the players are induced to cooperate by helping each other to identify as many target objects as possible: Only if each player collects all his/her own target objects, the whole team succeeds. In contrast, while playing in competitive mode, the player that collects the highest number of target objects wins. In both the collaborative and the competitive scenarios, the players can interact via voice chat, with this type of communication being hands-free and comfortable even while cycling. Obviously, communicating is particularly relevant in the collaborative scenario, in which players should give each other suggestions about the approaching target.

### 3.1. SocialBike Architecture

SocialBike is a PC-based application whose main hardware components are: A cycle ergometer (Cycle Ergometer 100 K, COSMED, Rome, Italy), a pushing button anchored on the cycle ergometer handlebars, and an Arduino2 board connecting the button to the computer. The cycle ergometer must be placed in front of a screen that allows for the displaying of the VR scenarios. Larger screens, such as TVs or projected screens, are preferred to smaller ones to facilitate the correct identification of the appearing target objects. 

To measure the heart rate, two different possibilities have been implemented. The first foresaw the use of a commercial sensor mounted on a chest band (Polar H1), while the second is based on a finger pulse oximeter that can be connected to the same Arduino2 board that handles the detection of the button push. These two solutions are interchangeable and can be selected according to the trainee’s preference or specific indications from clinicians (e.g., the correct positioning of the chest strap is more difficult than the pulse oximeter application, and therefore the pulse oximeter may be indicated more for weaker users).

**Figure 2:**
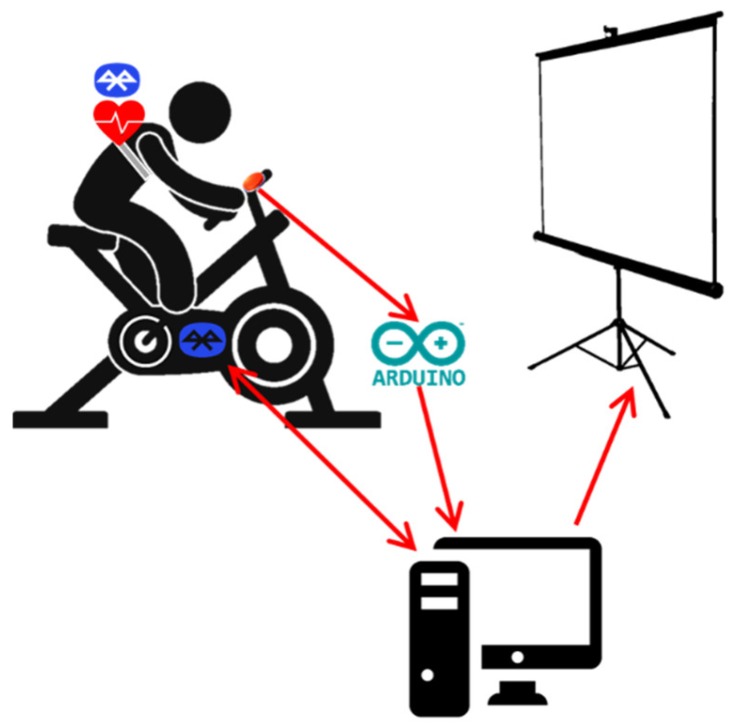
The system configuration. In this case, the user is wearing a chest band equipped with a sensor and a Bluetooth module for the communication with the cycle ergometer controller.

For the measurement of heart rate using the chest band, as well as to set and measure the cycle ergometer workload and velocity, an ad hoc communication protocol has been developed that exploits the ergometer Software Development Kit (SDK) provided by the manufacturer. The stream of heart rate data occurring between the sensor and the cycle ergometer is managed by an adapter, which communicates with the Polar device via Bluetooth, as illustrated in Figure 2. 

The cycle ergometer provides the measurements of the following quantities in real time: Revolutions per minute (RPM), workload, and time elapsed. Linear velocity is calculated in the software application as *v* = r × ω, where r is the estimated wheel radius and ω the angular velocity (in RPM).

For the identification of the button push and for the eventual measurement of the user’s heart rate via pulse oximeter, a second communication protocol allowing for a data stream through the serial port has been implemented.

The virtual environments have been developed using Unity 3D [55]. Photon Unity Networking (PUN) and Photon Voice have been used for the implementation of the multiplayer features and the voice chat, respectively. Both these Unity packages are based on Photon Engine [56], a cloud platform that hosts games over the internet, enables ubiquitous access to shared resources, and guarantees low latency for the exchange of information among the different players.

#### Application Networking

Photon Cloud is built using a room-based architecture, which means that players are aware of each other’s presence only if they are located in the same ‘shared environment’, i.e., the ‘room’. Each room is independent from the others and is managed by a specific ‘game server’. 

Rooms are organized inside ‘lobbies’, which represent virtual containers for which the developer can set and retrieve several room properties. Non-random matchmaking among players can be eased by specifying the lobby to join, instead of using the default one.

As already mentioned, data sharing among players can occur only if they are located in the same room. There are three different methods to handle network messages:

- The continuous streaming of data across the network (e.g., to communicate to others the current position and rotation of a specific player), performed by a serializing function.
- ‘Remote Procedure Calls’ (RPCs) for infrequent actions.
- The change in game objects’ ‘Custom Properties’, for very rare status updates.

Photon also allows for the implementation of a voice chat. This feature is fundamental in the SocialBike application, since all the players have to communicate hands-free to set up exercise groups and, in particular, to collaborate for the identification of the targets appearing along the path. The functionalities needed to implement the voice chat are contained in the Photon Voice add-in. This package handles the connection workflow, focusing on audio transmission. 

### 3.2. The Application Flow

**Login.** The experience of using the SocialBike application starts with the user login. Each user has a predefined profile properly stored in a Resource Description Framework/eXtensible Markup Language (RDF/XML) file. The user profile contains both personal information and data regarding the training sessions (e.g., number of sessions performed, date, time, and score of each session). 

New users can be created by accessing a password-protected area of the software, which should be accessed by clinicians or developers before delivering the application to the end users’ homes. As already mentioned, the user’s birth date is a particularly relevant piece of information because the calculation of the target heart rate (HR) value is computed as 65% of the maximum frequency, which is estimated according to the subject’s age. In case of a particularly frail individual, the standard value of 65%, which is suggested in literature to elicit a moderate effort in elderlies [57], can be modified by specifying an alternative percentage in the user’s profile.

During the login, the user is connected to the Photon Network and joins a customized lobby, and then the ‘Waiting Room’. If no other players are online, the user should wait in this room for others to connect.

**The Waiting Room**. This room constitutes the entry point of the social application. In this room, in fact, all the users who are online (but still not exercising) can meet and talk to each other either to set up teams for the collaborative game, or to find rivals for the competitive scenarios. Each user is identified by a customizable icon, his/her nickname, and his/her unique identifier. When a user is talking, his/her icon is highlighted so that all the others are aware of who he or she is.

**Figure 3:**
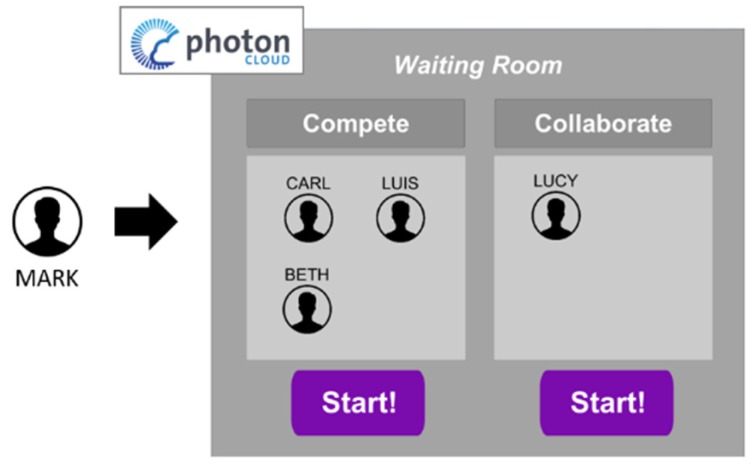
A schematic representation of the application interface. The user ‘Mark’ logs in and joins the ‘Waiting Room’ in which other players are already present.

**Figure 4:**
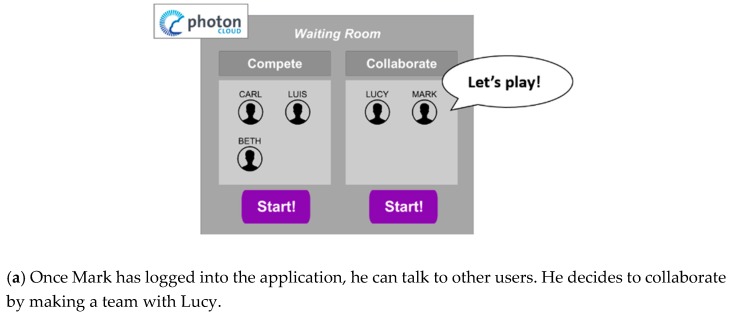

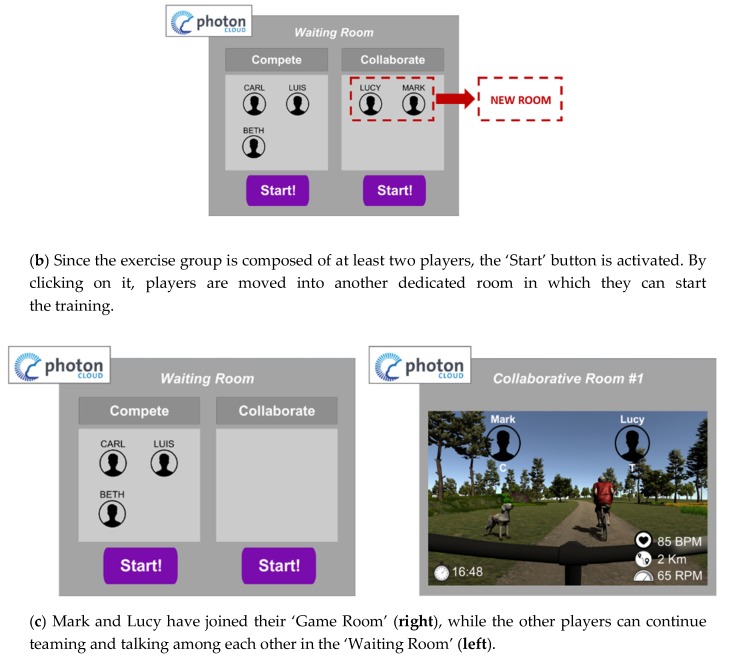
An exemplificative representation of the application flow.

While being in this room, the users have the chance to setup the exercise groups by clicking on the ‘Collaborate’ or on the ‘Compete’ button, as shown in Figure 3. To avoid confusion during the exercise, each group can be composed of a maximum of four players. To ease the comprehension of the group creation, each time a player chooses their game mode, his/her icon is moved into a 2 × 2 grid-based panel, as illustrated in Figure 4a. 

If at least two players have chosen the same game mode, the ‘Play’ button activates and they can start exercising.

In the ‘Waiting Room’, the players’ icons’ instantiation and placement in one of the two panels are handled using RPC. The beginning of the exercise is marked by the creation and the joining of a dedicated ‘Game Room’. ‘Game Rooms’ are accessible only to the players who have already chosen to play in that specific mode at the ‘Start’ button click, i.e., those whose icons were present in the collaborative or competing panel, for example Lucy and Mark in Figure 4b.

**Pre-training.** When all the players are in the ‘Game Room’, either to compete or to collaborate, they are given a brief explanation on how to perform the exercise i.e., maintain the cycling velocity between 60 and 70 RPM and push the button when the target animal or object appears. They are also asked to wear the HR sensor and to sit comfortably on the cycle ergometer through the provision of visual text instructions.

When the application detects the heart rate measurement, each player is randomly assigned the *target* (i.e., a letter or a color), and the *order*, based on the player’s actor ID (index identifying a player in a room), which determines the position of the path a specific player will follow during the cycling.

Then, every player communicates when he/she is ready to begin by pushing the button on the cycle ergometer. This information is conveyed to the other players through the User Interface (UI), where a small image with the nickname and the assigned *target* appears.

The *target* animals or objects and the *distractors* are instantiated in the scene at specific positions either at the right or left side of the pathway. The positions are generated randomly and then communicated to the other clients using a RPC, which triggers the instantiation of the *targets* in the same position in each player’s scene. In this way, the positions of the *targets* remain fixed for all the players sharing the same ‘Game Room’ but differ from one room to another, allowing for an infinite number of possible configurations.

When all the players are ready, a 10 s countdown starts.

**Figure 5:**
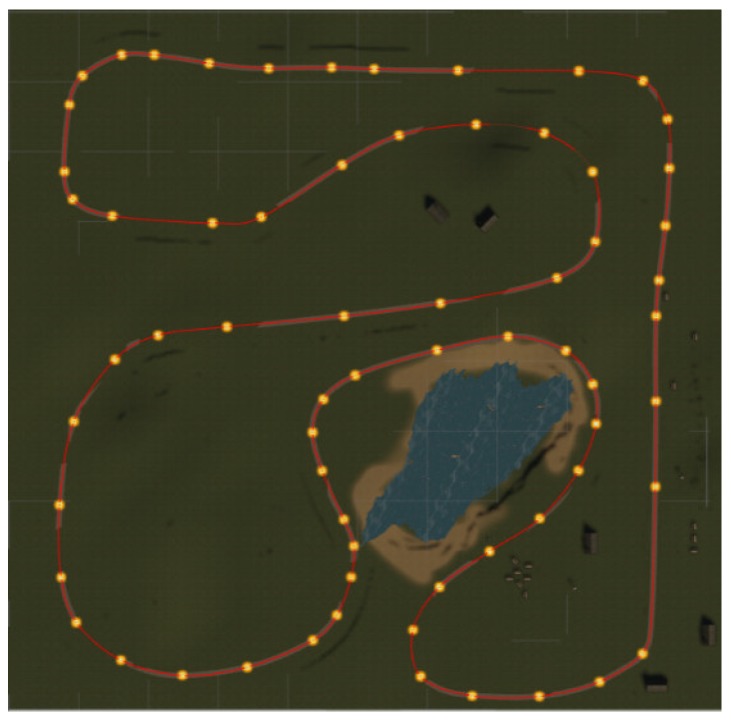
One of the paths (red line) viewed from the top. Each path is constituted by a series of nodes (highlighted in orange) that are interpolated in real time. Other paths, dedicated to other users, run in parallel.

**Dual Task training.** During the exercise, data on the cycling velocity of each player are sent over the network so that all the users can see the others’ avatars moving. Each player is represented by a cycling avatar, which, up to now, is not customizable with the exception of gender. The synchronization of position and rotation is handled through a continuous stream of data across the network. Each player moves along their own lane according to the real cycling speed, measured from the cycle ergometer. Each path is generated by a series of nodes placed along the route, as illustrated in Figure 5, whose interpolation occurs in real time using quaternion spherical linear interpolation (slerp). The user cannot brake or turn intentionally, and the intent of reducing sharp bends as much as possible was pursued to avoid the occurrence of cyber-sickness due to the expectation of lateral accelerations [58].

Since the path is not following a linear trajectory, a correction factor is applied to the translation so that all the players travel the same distance as the player in the shortest lane (order = 1). Data coming from the HR sensor, as well as the ergometer workload, are kept private and are processed by each single client independently.

With respect to the cognitive task, every time a player pushes the button, there are three possible situations: (1) the player is close to a *target*, (2) the player is close to a *distractor*, or (3) there is no object close to the player. The concept of “being close” is defined by the area covered by a collider, shaped as a truncated square pyramid, with the smaller base matching the head of the player. Such a collider simulates the player’s field of view (FOV) so that if a collision with the object occurs (i.e., the object falls within the player’s FOV), then the button click corresponds to the selection of the target or the distractor objects. Otherwise, the player falls into situation (3).

**Figure 6:**
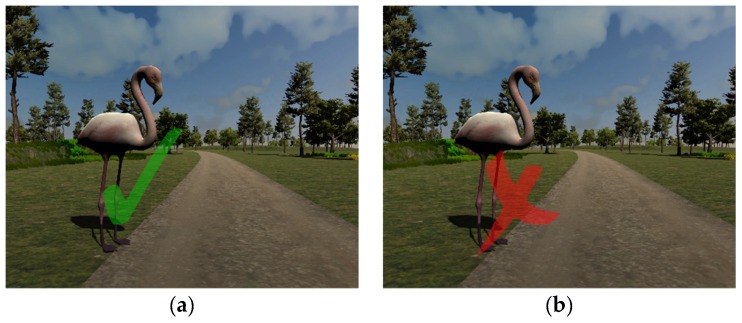
Examples of correct (**a**) and wrong (**b**) choice feedback to a user’s selection.

The player’s action is sent over the network only in situation (1). In this case, the player’s score is updated through RPCs so that all the other participants are aware that a specific player has successfully collected one of his/her targets. The task does not include penalties for players who make a wrong choice. Instead, this information is kept private and stored only at the client level. However, each player gets visual feedback on his/her own actions: Every correct or wrong choice is displayed as a green check or a red x-cross, respectively, appearing near the related object as shown in Figure 6.

All the useful data are visible to each player through the Graphical User Interface (GUI). The values of heart rate and cycling speed are displayed privately, so that each player gets real-time feedback on his/her own physical performance. Proper messages to alert the user in case the HR exceeds safety levels, or to suggest slowing down or speeding up if he/she is cycling too fast (>70 RPM) or too slow (<60 RPM) are also shown privately. In this case, a penalty (i.e., score decreased by 0.5) is due for the players that, 30 s after the alert, have not brought their speed back to an acceptable value.

The performance in the cognitive task is represented by a score, which is visible to all the players in the same way: As the sum of the *targets* collected by each player (collaborative mode) or as the number of *targets* already collected by each player, identified by nickname (competitive mode). In addition, all the players can see the time remaining to accomplish the task.

**Report of the training.** At the end of the training, a report containing all the data of the session is produced. Data include shared information about the multiplayer mode (collaborative or competitive), the number of players, their nicknames, *targets*, and *orders*. The same report also contains the private data regarding the single player’s performance in both the physical and cognitive tasks. In particular, heart rate, workload, and cycling speed are saved every 4 s. The performance in the cognitive task is represented by the user’s action, which is saved every time he/she gets close to a target object. The action may be: “correct” (the user collected a target), “wrong” (the user collected a distractor), or “no interaction” (the user did not perform any action). For every target, the name, the type, the feature to identify (i.e., the initial letter or the color), the position (ID node), and the user’s action are stored. Moreover, the player’s final score and the total score (if collaborative) or the single players’ scores (if competitive) with the corresponding players’ nicknames are saved.

All these pieces of information may be shared with other players and relatives using an ad hoc social media network.

## 4. SocialBike in the “House of the Future”

### 4.1. Social Media Network

For the effective exploitation of SocialBike at home, the presence of a framework allowing for the communication, mates finding, and the exchange of information among all the possible players is essential. To do this, SocialBike has been implemented into a social media network (SMN) specifically developed to encourage inclusion among elderlies, thus reducing the risk of isolation. The SMN will be developed by leveraging the capabilities offered by the Semantic Web technologies [59], which are a promising tool both in the fields of AAL [60] and in the description of social networks [61]. Leveraging ontologies (i.e., explicit specifications of a conceptualization) [62] makes possible to represent the users and their characteristics, and to match them according to specific features. The SMN also allows for the representation of the *status* of a user—namely if he/she is in a Waiting Room to find a rival (in a competitive session) or to find a partner (in a collaborative session).

**Figure 7:**
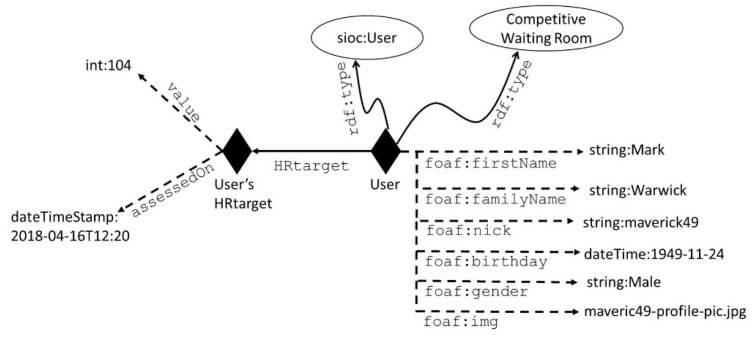
An example of user’s representation in the SocialBike social media network (SMN). Individuals are represented with diamonds, concepts are represented with circles, and roles are represented with arrows (dashed arrows indicate datatype properties, while full-line arrows represent object properties). The type of an individual is stated with a curved arrow.

For the SocialBike SMN, a few existing vocabularies have been reused to describe the users and their features, together with some new classes and properties dedicated to representing relevant pieces of knowledge for SocialBike. The Friend Of a Friend ontology (FOAF) [63], which refers to a set of people-related terms that allows the description of the relations among individuals in a machine-readable way, and Semantically-Interlinked Online Communities (SIOC) (a vocabulary providing the main concepts for information description in online communities) have been partially adopted to describe the users and their information. Both FOAF and SIOC are developed with XML-compliant formats (RDF/XML and RDF/ Web Ontology Language (OWL), respectively) and can be used to interpret and publish information on web pages. FOAF can be used in the form of structured data, such as RDFa (Resource Description Framework in attributes) [64], and can be included into web pages. In this way, the SMN can be enjoyed via both desktop and mobile navigation experiences. SIOC is adopted to provide formal definitions regarding the interactions among the users. Figure 7 provides an example of user modelling within the SocialBike SMN.

The SocialBike SMN’s ontological layer allows for information interchange among users and the results they achieve in both competitive and collaborative sessions. It can also suggest mates for both collaborative and competitive sessions, leveraging on the representation of some of the dwellers’ health-related data (i.e., age and HR_target_) and on matching rules developed with Semantic Web Rule Language (SWRL) [65]. Therefore, by exploiting the reasoning capabilities provided by semantic technologies and on the possibility to describe the *status* of a user (i.e., whether he/she is in a Waiting Room for a collaborative or competitive session), the SocialBike SMN enables the receiving of notifications indicating which other users could be interested in joining a team for collaborative sessions, thus improving the chances for social connection among the players.

**Figure 8:**
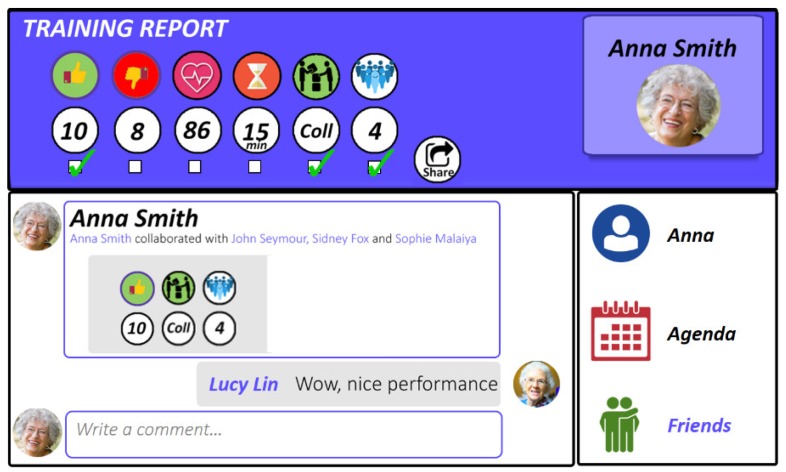
A screenshot of the SocialBike SMN. The user (Anna) can choose which data can be seen by her friends when sharing her performance, and manage her personal data, her agenda, and her friends’ list.

The SMN is designed to be essential in its graphical user interface, and provides some features dedicated to enhance the SocialBike experience, as illustrated in Figure 8.

Each user can specify some personal data (such as full name, location, birth date, and contact details) and create his/her own personal page, which can be personalized by adding a picture or a snapshot of the avatar used to the SocialBike application. The main aims of the SMN are: Allowing a player to find new teammates and/or competitors; sharing his/her results (or a part of the results achieved); and scheduling a collaborative session with the teammates or to state his/her own availability for a session. These aims can be achieved by leveraging specific SIOC’s properties (e.g., sioc:has:reply, sioc:reply_of) and classes (e.g., sioc:Post and sioc:Thread), thus providing a semantic representation of users’ interactions on the SocialBike SMN. To foster offline in-person socialization, the SocialBike SMN also takes into account users’ localizations in the world, using the GeoNames ontology [66] to describe the inhabitant’s location (i.e., the city where he/she lives in). In this way, users collaborating with or competing against each other in the virtual world can meet in the real world, thus promoting active participation in the social life, and a better quality of life.

The SocialBike SMN’s notifications and alerts, such as when a competitor or a collaborator is available for a session or when a new friend request is pending, are received via the HIC and shown, respectively, in the *Agenda* or *Friends* sections. When the inhabitant accepts an invitation for a competitive or collaborative session, he/she can start the SocialBike application, wear the HR sensor, and then start the actual exercise session as described in Section 3.2.

### 4.2. User’s Monitoring and Progress

**Figure 9:**
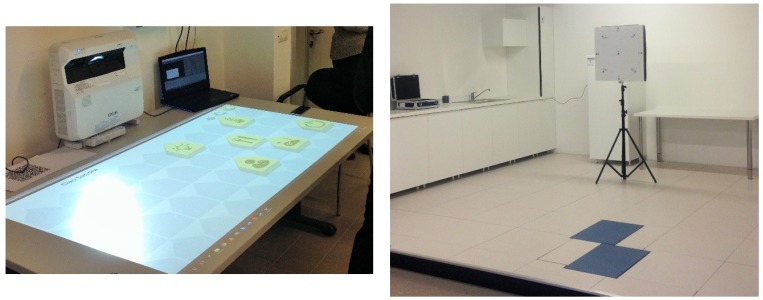
A picture of Home Interactive Controller (HIC) interface projected on a table (**left**), and Future Homes for Future Communities (FHfFC) Living Lab (**right**); in blue, on the floor, are the two force platforms.

Besides the equipment dedicated to the performance of the exercise itself and the already mentioned Home Interactive Controller (HIC) enabling the control of all the house metrics and actuations, as shown in Figure 9 (right), the FHfFC living lab is also equipped with two force platforms (Biomechanics Force Platform, AMTI, Watertown, MA, USA) that allow for the measurement of the user’s postural sway. These are also shown in Figure 9 (right).

The term “postural sway” refers to the corrective body movements resulting from the control of body position [67]. Sway is evaluated during upright standing through the measurement of body center of pressure (COP) excursion. In healthy subjects, balance during stance is maintained as long as COP oscillates within the base of support (i.e., the area beneath a person that includes every point of contact that the person makes with the supporting surface). Therefore, COP excursion is used as a measure of the body’s effort at maintaining balance in that posture, with increased sway indicating greater effort, and thus increased risk of falling.

Different studies have highlighted how COP oscillations, particularly in the medio-lateral (M–L) direction [67], and covered ellipse area [68] can be correlated with reduced body stability, and thus poorer balance. Therefore, measuring COP excursions at regular intervals during the training period could represent an effective way of monitoring an elderly’s improvements in balance over time, even in an unsupervised setting such as the home. Indeed, for the real home implementation, even a mobile force platform does not represent an affordable solution. However, it has been proven that cheaper solutions, such as the Wii Balance Board, could provide reliable measures, thus allowing for the measurement of postural sway in home-based settings too [69].

For all these reasons, postural sway (i.e., anterior–posterior and M–L displacement and covered ellipse area) will be included, as an objective measure, in the protocol for the validation of the DT training. This piece of data, collected thanks to an appropriate force sensor and through a reminder given to the user via the HIC, will be stored in the user’s ontology-based profile, together with other already mentioned session- and health-related data.

In the future, the integration of newly-introduced methods for the evaluation of postural sway, such as the time-integrals of displacement (absement, absity, abseleration, etc.) [70,71], will be evaluated to provide a more in-depth evaluation of the effectiveness of the DT-based treatment we have designed.

## 5. Conclusions

This work presents an application that provides frail elderlies with a Dual Task training program at home. The final aims are reducing the risk of falling, through the improvement of the clinical outcomes of frailty, and promoting their social participation.

The proposed exergame, based on a previous work developed in a clinical environment [17], exploits a setup that can be easily and safely implemented in a home-based setting. The VR-based social application allows the users to select the way in which they prefer to train, thus enhancing the motivation to practice by proposing to collaborate or compete according to each user’s personality and attitude.

The application for single players is being validated in the Istituto Auxologico Italiano, enrolling a sample of frail elderly in a supervised setting. These tests are aimed at assessing the feasibility of the intervention, the acceptability of the technology, and the levels of engagement and enjoyment elicited by a DT training program involving a cycling task and a VR-based cognitive activity. Preliminary results, obtained by enrolling a sample of five elderlies and using a slightly different setup involving a CAVE, showed a good-to-excellent usability (System Usability Scale, SUS = 76.88 ± 17.00) and high levels of flow (Short Flow Scale = 4.33 ± 0.75) [72]. The study also highlighted some aspects that needed to be enhanced, e.g., targets’ recognizability and placement, and sound meanings. These features have all been improved according to both elderlies’ and therapists’ suggestions [72]. Therefore, given the encouraging results already obtained, we believe that the social version of the system could also be exploited successfully. Once the validation in the clinical setting has ended, further tests investigating the effect of the social part will be performed, in the clinic and/or at elderlies’ homes.

Regarding the software application, particular attention will be paid to the customization of avatars during the next months. In this first prototype, in fact, there is no possibility of modifying the physical features of the cycling avatar. Instead, it just serves as a reference to know where the other players are and to adapt the velocity to proceed together. However, some studies showed that having the possibility to customize some features may positively influence embodiment, and thus motivation to practice [73,74]. Therefore, it appears interesting to evaluate if the customization of some avatars’ characteristics, perhaps through a reward mechanism, enhances the participation and the engagement of the elderly.

Finally, before using SocialBike at home, another important aspect related to the fruition of the service should be taken into consideration. In fact, it will be necessary to evaluate the real costs of the final solution through a proper business model, and eventually to implement a rental mechanism for the provision of the rehabilitative equipment to each end user’s house.
